# Sex differences in the association between chest computed tomography-defined sarcopenia and cardiovascular risk factors among inpatients

**DOI:** 10.3389/fnut.2024.1431036

**Published:** 2024-09-02

**Authors:** Xin Chen, Mingyu Zhu, Jie Cao, Didi Zuo, Zengai Chen, Yurong Weng, Hua Jiang, Yaomin Hu

**Affiliations:** ^1^Department of Geriatrics, Shanghai East Hospital, School of Medicine, Tongji University, Shanghai, China; ^2^Department of General Practice, Shanghai East Hospital, School of Medicine, Tongji University, Shanghai, China; ^3^Department of Geriatrics, Renji Hospital, School of Medicine, Shanghai Jiaotong University, Shanghai, China; ^4^Department of Radiology, Renji Hospital, School of Medicine, Shanghai Jiaotong University, Shanghai, China

**Keywords:** sarcopenia, chest CT, skeletal muscle area, cardiovascular diseases risk factors, inpatients

## Abstract

**Background:**

While sarcopenia has been found to be associated with increased risks of cardiovascular diseases (CVDs), evidence exploring sex-related differences remains insufficient. This study aimed to investigate the differences in how often sarcopenia occurs in each sex, as determined by skeletal muscle area (SMA) in chest CT images, and its association with CVD common risk factors.

**Methods:**

This cross-sectional study involved 1,340 inpatients from the Department of Geriatrics of Renji Hospital, affiliated to Shanghai Jiaotong University School of Medicine. Data on age, sex, body mass index (BMI), smoking status, disease history, and clinical parameters were collected. Sarcopenia was defined using chest CT images with a cut-off value of T12-SMA/height^2^ <25.75 cm^2^/m^2^ in male patients and <20.16 cm^2^/m^2^ in female patients. Cardiovascular risk was assessed using the Framingham risk score (FRS). The association between T12-SMA/height^2^-defined sarcopenia and CVD risk factors by sex was evaluated using a multivariate logistic regression analysis.

**Results:**

The overall prevalence of T12-SMA/height^2^-defined sarcopenia (<25.75 cm^2^/m^2^ for male patients, <20.16 cm^2^/m^2^ for female patients) was 54.03%, with 48.09% in male patients and 63.19% in female patients. The proportion of male patients with high CVD risk was greater than that of female patients. The multivariate analysis revealed that T12-SMA/height^2^-defined sarcopenia was independently associated with age (in male patients only), systolic blood pressure (SBP), cholesterol, and high-density lipoprotein cholesterol (HDL-C) among the six FRS cardiovascular risk indices.

**Conclusion:**

Our results suggest that T12-SMA/height^2^-defined sarcopenia was more prevalent in male patients than in female patients. Sarcopenia was associated with higher levels of SBP and HDL-C and lower levels of cholesterol. Increasing age had a more significant effect on CVD risk in male patients.

## Introduction

Sarcopenia is a progressive and generalized condition characterized by the degeneration of skeletal muscle, resulting in reduced muscle mass and function (1, 2). Among inpatients at nutritional risk, the prevalence of sarcopenia can reach as high as 68.4% (3). This condition has been proven to be associated with multiple adverse outcomes, including falls, physical disability, functional decline, frailty, and mortality (4). Moreover, sarcopenia has been proposed to be associated with increased risks of cardiovascular diseases (CVDs) and may accelerate the progression of CVD, especially in middle-aged and older adults (5).

CVD is the leading cause of death globally. The prevalence of sarcopenia among patients with heart failure ranges from 34 to 66%, with the highest rates observed in patients hospitalized for acute decompensated heart failure (6). In addition, reduced skeletal muscle mass has been proven to be associated with increased coronary artery calcium scores, subclinical atherosclerosis, arterial stiffness, and thickening of the carotid arterial wall in asymptomatic older adults (7). Given these associations, it is important to identify CVD risk factors in sarcopenia patients.

The diagnosis of sarcopenia is still evolving. While assessing whole-body muscle mass using a whole-body computed tomography (CT) scan is considered accurate, it is not practical for routine use. The third lumbar vertebra (L3) in abdominal CT scans is commonly used as an optimal CT axial image level for estimating whole-body muscle mass. However, chest CT scans are more commonly used in clinical practice, and growing evidence suggests that the cross-sectional skeletal muscle area (SMA) at the 12th thoracic vertebra (T12) level can also be used to diagnose sarcopenia (8–10). However, most clinicians are still unaware of this assessment and the diagnostic method for sarcopenia.

Considering sex as an important biological variable can enhance the identification of individuals at risk of CVD and facilitate more effective prevention and treatment strategies (11). Sarcopenia is generally more prevalent in female individuals than male individuals. However, there is limited evidence regarding potential sex-related differences in the association between sarcopenia and the most common risk factors for CVD. Therefore, the present study aimed to assess the prevalence of sarcopenia, as defined by chest CT images, in a large sample of hospitalized patients, evaluating both the overall prevalence and differences by sex. Additionally, the study sought to evaluate the association between sarcopenia and common CVD risk factors.

## Methods

### Study design

This was a retrospective cross-sectional study conducted on inpatients from the Department of Geriatrics of Renji Hospital, affiliated to Shanghai Jiaotong University School of Medicine. Data were collected between May 2021 and August 2023. The inclusion criteria for participants were as follows: (1) age between 30 and 75 years; (2) those who underwent a chest CT scan; and (3) those who provided informed consent. The exclusion criteria were as follows: (1) hospitalization for angina, acute myocardial infarction, stroke, transient ischemic attack, or peripheral vascular disease and (2) incomplete data. This study was approved by the Ethics Committee of Renji Hospital, affiliated to Shanghai Jiaotong University School of Medicine (KY2021-143-B).

### Assessment of sarcopenia status

Chest CT scans were performed using two multidetector CT scanners (Revolution 256 and Lightspeed 64; GE Healthcare) with a 1-mm slice thickness. Acquisition parameters were set according to the manufacturer's standard recommendations. Hounsfield unit (HU) values between −29 and +150 HU were used to semi-automatically delineate muscle areas, which were quantified using Slice Omatic software V4.3 (TomoVision, Montreal, Canada). The cross-sectional areas of the skeletal muscles (including the erector spinae, rectus abdominis, latissimus dorsi, obliquus externus, internus abdominis, and internal and external intercostal muscles) at the T12 pedicle level of the chest CT were measured as T12-SMA. To adjust for body size, the SMA was divided by the square of the body height (m^2^) (12). Sarcopenia was defined as T12-SMA/height^2^ < 25.75 cm^2^/m^2^ in male patients and < 20.16 cm^2^/m^2^ in female patients (10).

### Assessment of cardiovascular risk

CVD risk was assessed using the Framingham risk score (FRS), one of the most widely recognized assessment tools for estimating the 10-year risk of CVD in individuals aged between 30 and 74 years. The FRS calculation incorporates factors such as age, sex, total cholesterol, high-density lipoprotein cholesterol (HDL-C), systolic blood pressure (SBP), smoking habits, and diabetes status. Based on the calculated FRS value, individuals were categorized into three risk levels in accordance with the 2021 Canadian Cardiovascular Society dyslipidemia guidelines (13): low risk (< 10%), medium risk (10 to < 20%), and high risk (>20%).

### Data collection

Data were collected from electronic medical records. The following variables were collected: age, sex, body mass index (BMI), smoking status, disease history (including hypertension, diabetes, and dyslipidemia), systolic blood pressure (SBP), diastolic blood pressure (DBP), fasting plasma glucose (FPG), triglycerides (TG), total cholesterol (TC), high-density lipoprotein cholesterol (HDL-C), low-density lipoprotein cholesterol (LDL-C), and T12-SMA. Using standard procedures, venous blood samples were drawn after 12 h of fasting to determine FPG, TG, TC, HDL-C, and LDL-C levels.

Three consecutive blood pressure measurements were taken at 1-min intervals, with the mean values calculated and used for analysis. Age, BMI, height, disease history, and smoking status were recorded upon admission. Smoking was defined as having smoked at least 100 cigarettes in one's lifetime.

### Statistical analysis

All statistical analyses were conducted using Statistical Package for the Social Sciences (SPSS) version 24.0 (IBM Company, New York, NY, USA). Continuous variables were expressed as means and standard deviations (± SD) and analyzed using a one-way analysis of variance (ANOVA). Categorical variables were expressed as frequencies and percentages (%) and were analyzed using the chi-squared test (χ^2^). Pearson's correlation coefficient was used to assess the association between continuous variables. The logistic regression analysis was conducted to calculate crude and adjusted odds ratios (OR) with corresponding 95% confidence intervals (CIs) for factors associated with T12SMA/height^2^-defined sarcopenia. Factors with a *p*-value of < 0.1 in the univariate analysis were adjusted in the multivariate analysis. The receiver operating characteristic (ROC) analysis was performed to evaluate the goodness of fit for logistic regression models. *P*-values of < 0.05 were considered statistically significant.

## Results

### Characteristics of patients

A total of 1,340 patients (813 were male individuals and 527 were female individuals) with a mean age of 54.05 ± 10.19 years were included in this study. The basic demographic and clinical characteristics of the participants are shown in Table 1. The mean value of T12-SMA/height^2^ was 23.51 ± 5.56 cm/m^2^, and a total of 724 (54.03%) patients met the criteria for T12-SMA/height^2^-defined sarcopenia. Among the participants, 395 patients (29.48%) were classified as having medium CVD risk, and 440 patients were classified (32.84%) as having high CVD risk, according to the FRS.

**Table 1:** Characteristics of all participants by sex.

	**All (*n* = 1,340)**	**Male (*n* = 813)**	**Female (*n* = 527)**	***P*-value**
Age (years)	54.05 ± 10.19	54.06 ± 10.01	54.03 ± 10.47	0.953
BMI (kg/m^2^)	24.24 ± 3.68	25.34 ± 3.65	22.5 5 ±3.04	< 0.001
Smoking (*n*, %)	189 (14.10%)	185 (22.76%)	4 (0.76%)	< 0.001
Hypertension (*n*, %)	397 (29.63%)	286 (35.18%)	111 (21.06%)	< 0.001
Diabetes (*n*, %)	190 (14.18%)	152 (18.70%)	38 (7.21%)	< 0.001
Dyslipidemia (*n*, %)	299 (22.31%)	205 (25.22%)	94 (17.84%)	0.002
SBP (mmHg)	128.23 ± 17.55	131.31 ± 16.14	123.47 ± 18.55	< 0.001
DBP(mmHg)	79.72 ± 10.68	82.17 ± 10.53	75.95 ± 9.78	< 0.001
FPG (mmol/L)	4.96 ± 1.55	5.09 ± 1.75	4.76 ± 1.16	< 0.001
TG (mmol/L)	1.70 ± 1.16	1.92 ± 1.23	1.34 ± 0.95	0.005
Cholesterol (mmol/L)	4.99 ± 1.11	4.92 ± 1.13	5.09 ± 1.05	< 0.001
HDL-C (mmol/L)	1.21 ± 0.37	1.09 ± 0.31	1.38 ± 0.38	< 0.001
LDL-C (mmol/L)	2.89 ± 0.86	2.86 ± 0.87	2.95 ± 0.84	0.054
T12-SMA (cm^2^)	66.92 ± 18.92	77.83 ± 15.13	50.09 ± 9.48	< 0.001
T12-SMA/height^2^ (cm^2^/m^2^)	23.51 ± 5.56	26.12 ± 4.93	19.47 ± 3.80	< 0.001
Sarcopenia	724 (54.03%)	391 (48.09%)	333 (63.19%)	< 0.001
**FRS group (** ***n*** **, %)**				
Low risk	505 (37.69%)	138 (16.97%)	367 (69.64%)	< 0.001
Medium risk	395 (29.48%)	278 (34.14%)	117 (22.20%)	
High risk	440 (32.84%)	397 (48.83%)	43 (8.16%)	

BMI, body mass index; SBP, systolic blood pressure; DBP, diastolic blood pressure; FPG, fasting plasma glucose; TG, triglycerides; HDL-C, high density lipoprotein cholesterol; LDL-C, low-density lipoprotein cholesterol; SMA, skeletal muscle cross-sectional area; FRS, Framingham risk score.

Compared with female patients, male patients had higher levels of BMI, SBP, DBP, FPG, TG, T12-SMA, and T12-SMA/height^2^ and lower levels of cholesterol and HDL-C (all *p* < 0.05). In addition, male patients exhibited higher rates of smoking, hypertension, diabetes, dyslipidemia, and high CVD risk but a lower rate of sarcopenia (all *p*-values of < 0.05; Table 1).

### Characteristics of patients with sarcopenia

A total of 724 patients (391 were male individuals and 333 were female individuals) met the criteria for T12-SMA/height^2^-defined sarcopenia. Compared to female patients with sarcopenia, male patients with sarcopenia had higher values for age, BMI, SBP, DBP, FPG, TG, T12-SMA, T12-SMA/height^2^, and FRS, along with lower levels of cholesterol, HDL-C and LDL-C (all *p* < 0.05). In addition, male patients had higher rates of smoking, hypertension, diabetes, dyslipidemia, and high CVD risk but a lower prevalence of sarcopenia (all *p*-values of < 0.05; Table 2).

**Table 2:** Characteristics of the participants with sarcopenia by sex.

	**All (*n* = 724)**	**Male (*n* = 391)**	**Female (*n* = 333)**	***P*-value**
Age (years)	54.78 ± 10.51	56.82 ± 9.83	52.40 ± 10.80	< 0.001
BMI (kg/m^2^)	22.27 ± 2.59	23.16 ± 2.53	21.23 ± 2.23	< 0.001
Smoking (*n*, %)	83 (11.46%)	82 (20.97%)	1 (0.30%)	< 0.001
Hypertension (*n*, %)	168 (23.20%)	118 (30.18%)	50 (15.02%)	< 0.001
Diabetes (*n*, %)	93 (12.85%)	78 (19.95%)	15 (4.50%)	< 0.001
Dyslipidemia (*n*, %)	123 (16.99)	72 (18.41%)	51 (15.32%)	0.268
SBP (mmHg)	124.68 ± 17.47	128.66 ± 16.14	120.02 ± 17.83	< 0.001
DBP (mmHg)	77.25 ± 9.94	79.31 ± 9.70	74.84 ± 9.68	< 0.001
FPG (mmol/L)	4.86 ± 1.53	5.09 ± 1.88	4.60 ± 0.91	< 0.001
TG (mmol/L)	1.38 ± 0.80	1.57 ± 0.87	1.16 ± 0.64	< 0.001
Cholesterol (mmol/L)	4.87 ± 1.08	4.75 ± 1.11	4.99 ± 1.03	0.003
HDL-C (mmol/L)	1.28 ± 0.39	1.15 ± 0.34	1.43 ± 0.39	< 0.001
LDL-C (mmol/L)	2.82 ± 0.86	2.77 ± 0.88	2.88 ± 0.84	0.08
T12-SMA (cm^2^)	56.03 ± 12.96	65.89 ± 8.81	44.45 ± 5.07	< 0.001
T12-SMA/height^2^ (cm^2^/m^2^)	19.78 ± 3.39	22.06 ± 2.69	17.09 ± 1.79	< 0.001
FRS group (*n*, %)				< 0.001
Low risk	330 (45.58%)	62 (15.86%)	268 (80.48%)	
Medium risk	189 (26.10%)	137 (35.04%)	52 (15.62%)	
High risk	205 (28.31%)	192 (49.10%)	13 (3.90%)	

BMI, body mass index; SBP, systolic blood pressure; DBP, diastolic blood pressure; FPG, fasting plasma glucose; TG, triglycerides; HDL-C, high density lipoprotein cholesterol; LDL-C, low-density lipoprotein cholesterol; SMA, skeletal muscle cross-sectional area; FRS, Framingham risk score.

### Characteristics of the study population by sex and across categories of T12-SMA/height^2^

Among male patients, 48.09% had T12-SMA/height^2^-defined sarcopenia (< 25.75 cm^2^/m^2^). Male patients with sarcopenia were older and had lower levels of BMI, SBP, DBP, TG, cholesterol, and LDL-C but exhibited higher levels of HDL-C (all *p* < 0.05). Additionally, they presented a lower prevalence of hypertension and dyslipidemia (all *p*-values of < 0.05). There was no difference in CVD risk assessed using the FRS between patients with sarcopenia and those without sarcopenia (*p* > 0.05; Table 3).

**Table 3:** Characteristics of the male and female participants by sarcopenia status.

	**Male (*****n*** = **813)**			**Female (*****n*** = **527)**	
	**Sarcopenia (*n* = 391)**	**No-sarcopenia (*n* = 422)**	***P*-value**	**Sarcopenia (*n* = 333)**	**No-sarcopenia (*n* = 194)**	***P*-value**
Age (years)	56.82 ± 9.83	51.51 ± 9.49	< 0.001	52.40 ± 10.80	56.82 ± 9.25	< 0.001
BMI (kg/m^2^)	23.16 ± 2.53	27.35 ± 3.35	< 0.001	21.23 ± 2.23	24.81 ± 2.91	< 0.001
Smoking (*n*, %)	82 (20.97%)	103 (24.41%)	0.243	1 (0.30%)	3 (1.55%)	0.112
Hypertension (*n*, %)	118 (30.18%)	168 (39.81%)	0.004	50 (15.02%)	61 (31.44%)	< 0.001
Diabetes (*n*, %)	78 (19.95%)	74 (17.54%)	0.378	15 (4.50%)	23 (11.86%)	0.002
Dyslipidemia (*n*, %)	72 (18.41%)	133 (31.52%)	< 0.001	51 (15.32%)	43 (22.16%)	0.048
SBP (mmHg)	128.66 ± 16.14	133.77 ± 15.76	< 0.001	120.02 ± 17.83	129.40 ± 18.32	< 0.001
DBP (mmHg)	79.31 ± 9.70	84.84 ± 10.58	< 0.001	74.84 ± 9.68	77.86 ± 9.67	0.01
FPG (mmol/L)	5.09 ± 1.88	5.09 ± 1.61	0.993	4.60 ± 0.91	5.04 ± 1.45	< 0.001
TG (mmol/L)	1.57 ± 0.87	2.25 ± 1.41	< 0.001	1.16 ± 0.64	1.66 ± 1.27	< 0.001
Cholesterol (mmol/L)	4.75 ± 1.11	5.07 ± 1.14	< 0.001	4.99 ± 1.03	5.25 ± 1.07	0.07
HDL-C (mmol/L)	1.15 ± 0.34	1.04 ± 0.28	< 0.001	1.43 ± 0.39	1.29 ± 0.35	< 0.001
LDL-C (mmol/L)	2.77 ± 0.88	2.93 ± 0.85	0.008	2.88 ± 0.84	3.06 ± 0.85	0.021
T12-SMA (cm^2^)	65.89 ± 8.81	88.89 ± 10.71	< 0.001	44.45 ± 5.07	59.77 ± 7.17	< 0.001
T12-SMA/height^2^ (cm^2^/m^2^)	22.06 ± 2.69	29.87 ± 3.28	< 0.001	17.09 ± 1.79	23.55 ± 2.71	< 0.001
FRS group (*n*, %)			0.697			< 0.001
Low risk	62 (15.86%)	76 (18.01%)		268 (80.48%)	99 (51.03%)	
Medium risk	137 (35.04%)	141 (33.41%)		52 (15.62%)	65 (33.51%)	
High risk	192 (49.10%)	205 (48.58%)		13 (3.90%)	30 (15.46%)	

BMI, body mass index; SBP, systolic blood pressure; DBP, diastolic blood pressure; FPG, fasting plasma glucose; TG, triglycerides; HDL-C, high density lipoprotein cholesterol; LDL-C, low-density lipoprotein cholesterol; SMA, skeletal muscle cross-sectional area; FRS, Framingham risk score.

Among female patients, 63.19% had T12-SMA/height^2^-defined sarcopenia (< 20.16 cm^2^/m^2^). Female patients with sarcopenia were younger and exhibited significantly lower levels of BMI, SBP, DBP, FPG, TG, and LDL-C but higher levels of HDL-C (all *p*-values of < 0.05). They also presented a lower prevalence of hypertension, diabetes, dyslipidemia, and CVD risk (all *p*-values of < 0.05; Table 3).

### Association between T12-SMA/height^2^-defined sarcopenia and CVD risk factors

**Figure 1:**
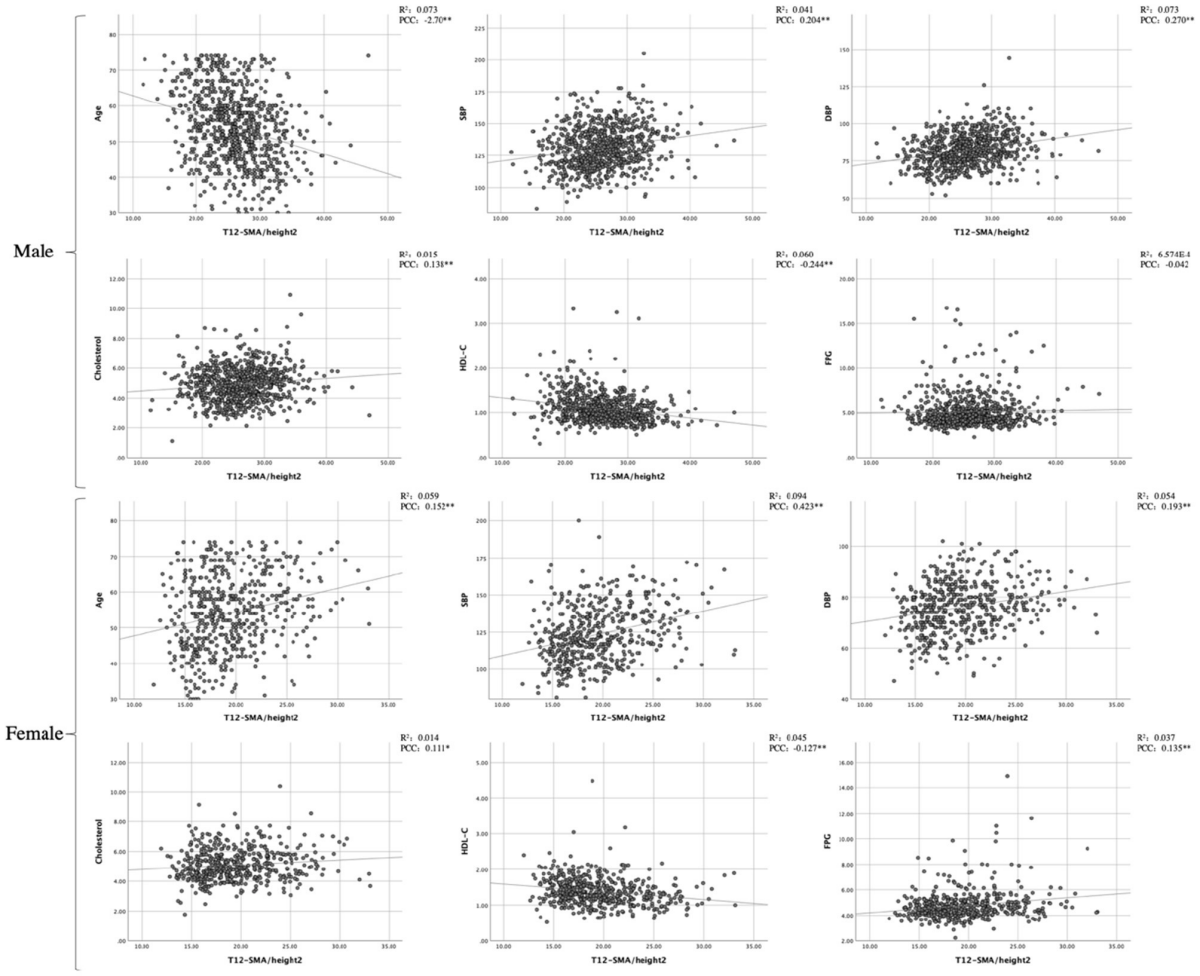
Pearson's correlation analysis and linear correlation analysis between CVD common risk factors and T12-SMA/height^2^. PCC, Pearson correlation coefficient; SMA, skeletal muscle cross-sectional area; SBP, systolic blood pressure; DBP, diastolic blood pressure; HDL-C, high-density lipoprotein cholesterol; FPG, fasting plasma glucose.

Pearson's correlation coefficient analysis revealed associations between T12-SMA/height^2^ and continuous variables. Among male patients, T12-SMA/height^2^ was negatively correlated with age and HDL-C and positively correlated with SBP, DBP, and cholesterol (all *p*-values of < 0.05). In female patients, T12-SMA/height^2^ was negatively correlated with HDL-C and positively correlated with age, SBP, DBP, cholesterol, and FPG (all *p* < 0.05; Figure 1).

**Figure 2:**
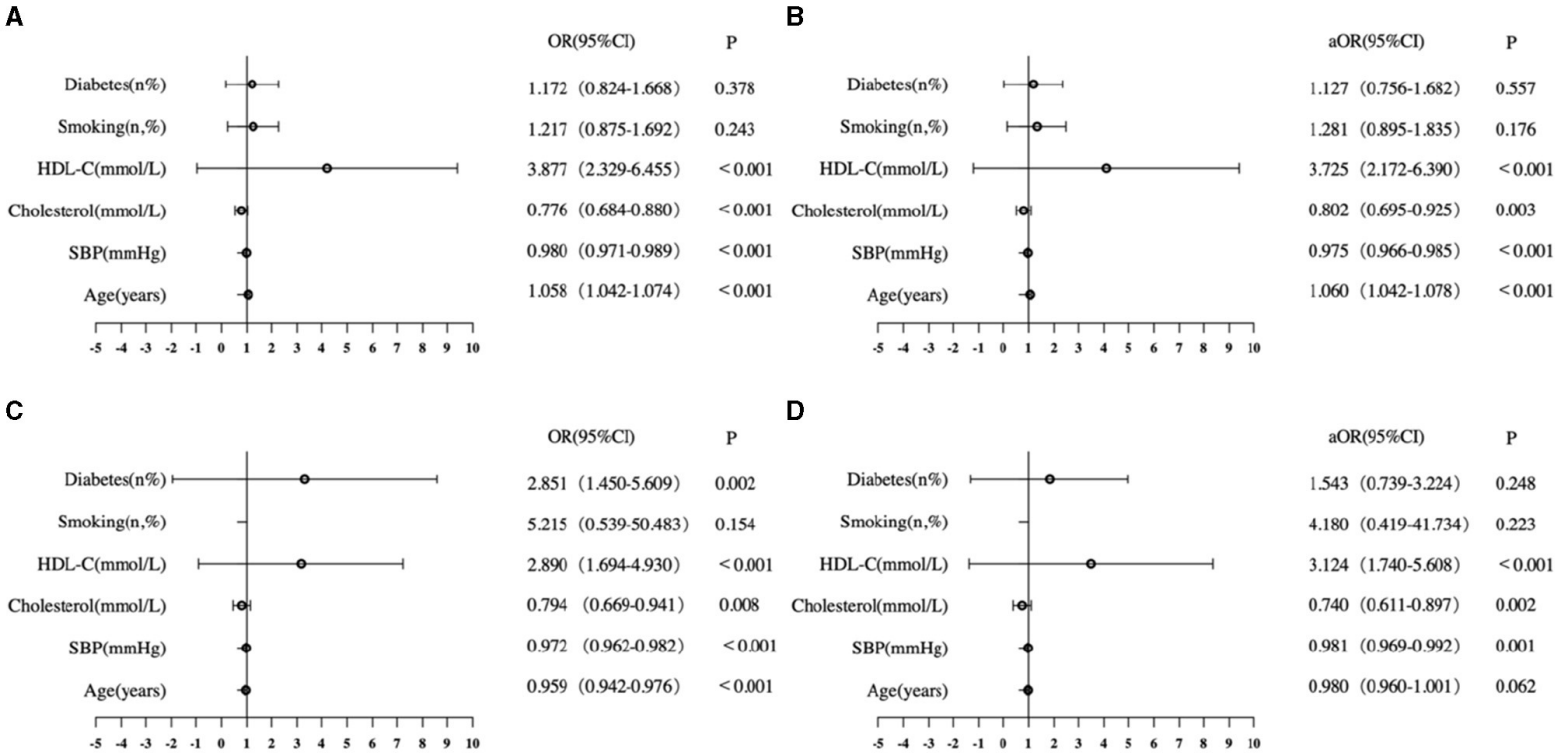
Logistic analysis of CVD common risk factors associated with T12-SMA/height^2^-defined sarcopenia. **(A)** Univariate analysis of male patients. **(B)** Multivariate analysis of male patients. **(C)** Univariate analysis of female patients. **(D)** Multivariate analysis of female patients. OR, odds ratio; CI, confidence interval; aOR, adjusted odds ratio; HDL-C, high-density lipoprotein cholesterol.

The logistic regression analysis identified six factors in the FRS that were associated with T12-SMA/height^2^-defined sarcopenia (< 25.75 cm^2^/m^2^ for male patients and < 20.16 cm^2^/m^2^ for female patients; Figure 2). In male patients, age, SBP, cholesterol, and HDL-C were associated with T12-SMA/height^2^-defined sarcopenia in the univariate analysis and remained independently associated with its multivariate analysis (all *p*-values of < 0.05; Figures 2A, B). In female, age, SBP, cholesterol HDL-C, and diabetes were associated with T12-SMA/height^2^-defined sarcopenia in the univariate analysis, but only SBP, cholesterol, and HDL-C were associated with T12-SMA/height^2^-defined sarcopenia in the multivariate analysis (all *p*-values of < 0.05; Figures 2C, D).

The ROC analysis showed that the model, which included age, SBP, HDL-C, cholesterol, smoking, and diabetes, had a high discriminative capacity for T12-SMA/height^2^-defined sarcopenia in both male (AUC = 0.716, 95% CI: 0.681–0.751, *p* < 0.001) and female (AUC = 0.711, 95% CI: 0.665–0.756, *p* < 0.001) patients.

## Discussion

This cross-sectional study revealed that 54.03% of inpatients presented T12-SMA/height^2^-defined sarcopenia (< 25.75 cm^2^/m^2^ for male patients and < 20.16 cm^2^/m^2^ for female patients), with a significantly higher prevalence in female patients (63.19%) than in male patients (48.09%). A greater proportion of male patients were at high risk for CVD compared to female patients. T12-SMA/height^2^-defined sarcopenia was independently associated with age (in male patients only), SBP, cholesterol, and HDL-C among the six FRS cardiovascular risk indices.

The prevalence of T12-SMA/height^2^-defined sarcopenia in this study was higher than that reported by most previous studies, which an estimated range of 13.85–49.83% (14–16). However, a secondary analysis of the Effect of Early Nutritional Support on Frailty, Functional Outcomes, and Recovery of Malnourished Medical Inpatients (EFFORT) trial showed that 68.4% of patients met the CT-based definition of sarcopenia (3). These differences could be attributed to varying inclusion criteria and definitions of sarcopenia. Generally, the prevalence of sarcopenia is lower in community-based populations compared to hospitalized patients (17, 18). This discrepancy may be attributed to the higher health status of the community population compared to hospitalized patients, who often have multiple comorbidities. In addition, sarcopenia can be diagnosed using various methods, such as bioelectrical impedance analysis (BIA), dual-energy x-ray absorptiometry (DEXA), dynamometer, typical gait speed measurements on a 6-m course (19), and abdominal CT scans. The use of different definitions of sarcopenia across studies can also contribute to variations in the reported incidence of sarcopenia.

We found that patients with sarcopenia had lower SBP than those without sarcopenia in both male and female patients, with no significant difference found in DBP. Pearson's correlation coefficient and the logistic regression analysis further confirmed that lower SBP was associated with sarcopenia. This finding suggests a possible vicious circle of low SBP and sarcopenia. Hypotension has been shown to inhibit muscle growth by causing impaired perfusion of muscle fibers (20). Similarly, the reduction of skeletal muscle in patients with sarcopenia leads to decreased venous return of blood to the heart due to muscle contraction, potentially resulting in hypotension.

However, most previous studies have reported results that are contrary to our findings, associating sarcopenia with higher DBP (21–23). These studies suggested that sarcopenia might induce inflammatory responses, leading to vessel wall thickening and increased blood pressure (24). The diversity in these results implies that both low and high blood pressure may be consequences of sarcopenia rather than causal factors. When muscle pumps are less effective at contracting veins, long-term impairment of venous return can occur, affecting blood pressure. The differences in the results across studies may be related to the different blood pressure ranges in the study populations.

Many sarcopenia patients also exhibit frailty, and previous studies have found a U-shaped association between SBP and mortality among frail participants (25, 26). Although many studies have suggested that sarcopenia is associated with hypertension (27, 28), it should be noted that aggressive antihypertensive therapy and poor health conditions that contribute to low blood pressure in sarcopenia patients may increase the risk of poor health outcomes (21).

In addition, sarcopenia was found to be negatively associated with cholesterol and positively associated with HDL-C in both male and female patients. This aligns with the findings of Jiang et al., who found that TC was negatively associated with sarcopenia in geriatric inpatients (14). They reported that an increase in TC within normal reference ranges may be a protective factor against sarcopenia, which is consistent with our results. An Italian study also demonstrated similar findings (29). Some studies, similar to the study of Wang et al., found high HDL-C levels in patients with sarcopenia, noting that HDL-C was positively associated with sarcopenia among community-dwelling Chinese adults (16).

However, a few studies have found low HDL-C levels in patients with sarcopenia. For instance, Habib et al. reported that TC was higher, but HDL-C was lower in sarcopenic obese individuals (30). This discrepancy may be due to the dynamics of lipid metabolism disorders. High levels of cholesterol contribute to the development and progression of atherosclerosis. HDL-C, with its anti-inflammatory and antioxidant properties, plays an important anti-atherogenic role. While HDL-C levels can be elevated within normal reference ranges to exert protective effects, sarcopenia may exacerbate dyslipidemia, leading to decreased HDL-C levels that trigger an inflammatory response, thereby potentially increasing cardiovascular risk.

This study also had some limitations that should be considered. First, the cross-sectional study design limited our ability to establish causal relationships between sarcopenia and CVD risk. Second, this study did not compare different diagnostic criteria for sarcopenia, which might impact comparisons with other studies. Third, the FRS was used to estimate CVD risk, but it did not account for genetic, environmental, and social factors. Therefore, future studies should consider longitudinal or randomized controlled designs to improve the quality of findings and develop new prevention strategies for sarcopenia, particularly in older people in clinical practice.

## Conclusion

Our results indicated that T12SMA/height^2^-defined sarcopenia is more prevalent in female individuals than in male individuals, and it is associated with higher levels of SBP and HDL-C and lower levels of cholesterol.

The use of chest CT scans to screen for the possibility of sarcopenia could reduce the number of required examinations, lower clinical costs, and offer good feasibility in clinical practice. While sarcopenia was significantly more prevalent in female individuals, the proportion of sarcopenic male patients with high CVD risk was higher. This finding suggests the need for increased vigilance regarding CVD risk in male patients with sarcopenia. In addition, the association between T12-SMA/height^2^-defined sarcopenia and age was significantly stronger in male patients, highlighting the importance of considering age-related impact on CVD risk in this group. Understanding how sex differences influence CVD risk factors in patients with sarcopenia can help clinicians in developing and correctly implementing more personalized prevention strategies.

## Data Availability

The raw data supporting the conclusions of this article will be made available by the authors, without undue reservation.
